# Clinical and multiparametric MRI features for differentiating uterine carcinosarcoma from endometrioid adenocarcinoma

**DOI:** 10.1186/s12880-024-01225-4

**Published:** 2024-02-19

**Authors:** Xiaodan Chen, Qingyong Guo, Xiaorong Chen, Wanjing Zheng, Yaqing Kang, Dairong Cao

**Affiliations:** 1 Department of Radiology, First Affiliated Hospital of Fujian Medical University, 20 Cha-Zhong Road, Fuzhou, 350005 Fujian P.R. China; 2 Department of Gynecologic Oncology, Fujian Maternity and Child Health Hospital, Affiliated Hospital of Fujian Medical University, Fuzhou, 350005 China; 3 Fujian Key Laboratory of Precision Medicine for Cancer, The First Affiliated Hospital, Fujian Medical University, Fuzhou, 350005 P.R. China; 4 Key Laboratory of Radiation Biology of Fujian Higher Education Institutions, The First Affiliated Hospital, Fujian Medical University, Fuzhou, 350005 P.R. China

**Keywords:** Magnetic resonance imaging, Uterine carcinosarcoma, Endometrioid adenocarcinoma, Diagnosis, Differential diagnosis

## Abstract

**Introduction:**

The purpose of our study was to differentiate uterine carcinosarcoma (UCS) from endometrioid adenocarcinoma (EAC) by the multiparametric magnetic resonance imaging (MRI) features.

**Methods:**

We retrospectively evaluated clinical and MRI findings in 17 patients with UCS and 34 patients with EAC proven by histologically. The following clinical and pathological features were evaluated: post- or pre-menopausal, clinical presentation, invasion depth, FIGO stage, lymphaticmetastasis. The following MRI features were evaluated: tumor dimension, cystic degeneration or necrosis, hemorrhage, signal intensity (SI) on T2-weighted images (T2WI), relative SI of lesion to myometrium on T2WI, T1WI, DWI, ADCmax, ADCmin, ADCmean (RSI-T2, RSI-T1, RSI-DWI, RSI-ADCmax, RSI-ADCmin, RSI-ADCmean), ADCmax, ADCmin, ADCmean, the maximum, minimum and mean relative enhancement (RE) of lesion to myometrium on the arterial and venous phases (REAmax, REAmin, REAmean, REVmax, REVmin, REVmean). Receiver operating characteristic (ROC) analysis and the area under the curve (AUC) were used to evaluate prediction ability.

**Results:**

The mean age of UCS was higher than EAC. UCS occurred more often in the postmenopausal patients. UCS and EAC did not significantly differ in depth of myometrial invasion, FIGO stage and lymphatic metastasis. The anterior-posterior and transverse dimensions were significantly larger in UCS than EAC. Cystic degeneration or necrosis and hemorrhage were more likely occurred in UCS. The SI of tumor on T2WI was more heterogeneous in UCS. The RSI-T2, ADCmax, ADCmean, RSI-ADCmax and RSI-ADCmean of UCS were significantly higher than EAC. The REAmax, REAmin, REAmean, REVmax, REVmin and REVmean of UCS were all higher than EAC. The AUCs were 0.72, 0.71, 0.86, 0.96, 0.89, 0.84, 0.73, 0.97, 0.88, 0.94, 0.91, 0.69 and 0.80 for the anterior-posterior dimension, transverse dimension, RSI-T2, ADCmax, ADCmean, RSI-ADCmax, RSI-ADCmean, REAmax, REAmin, REAmean, REVmax, REVmin and REVmean, respectively. The AUC was 0.997 of the combined of ADCmax, REAmax and REVmax. Our study showed that ADCmax threshold value of 789.05 (10^–3^mm^2^/s) can differentiate UCS from EAC with 100% sensitivity, 76.5% specificity, and 0.76 AUC, REAmax threshold value of 0.45 can differentiate UCS from EAC with 88.2% sensitivity, 100% specificity, and 0.88 AUC.

**Conclusion:**

Multiparametric MRI features may be utilized as a biomarker to distinguish UCS from EAC.

## Introduction

Malignant Mullerian mixed tumors (MMMTs) or malignant mesodermal mixed tumors were the previous names of carcinosarcomas. A tumor with both malignant mesenchymal and epithelial component is known as uterine carcinosarcoma (UCS) [1]. Being among the most malignant neoplasms to develop in the uterus, UCS are commonly misdiagnosed as endometrial carcinomas (EC) by dilatation and curettage or endometrial biopsy [2]. Due to the lack of distinct symptoms and clinical characteristics, such as vaginal bleeding and pelvic pain, the differential diagnosis of UCS and EC is challenging [3].

UCS has a 5-year survival rate of just 25% overall, which is much lower than EC [2, 4], while EC has an 83% 5-year survival rate [5]. Although UCS accounts for fewer than 5% of all uterine malignant tumors, it kills more than 16% of uterine cancer patients [6]. According to morphological appearances, a variety of imaging characteristics of UCS have been reported [2, 5, 7]. It is believed that the aggressiveness of UCS is caused by the grade of its adenocarcinoma [8]. Adenocarcinoma of endometrial origin is the epithelial component, according to histopathological analysis. The majority of initial cytological diagnoses are adenocarcinomas because the sarcomatous component adheres more than the adenocarcinoma component [2].

Immunohistochemical and molecular studies have suggested that the sarcoma component is in fact a metaplastic component derived from the carcinoma [9]. Therefore, UCS is expected to exhibit similar biological behavior to high-grade EC [9]. It has distinctive clinical and pathological characteristics which justify its separation from EC. UCS has a high incidence of lymphatic spread, peritoneal seeding, and lung metastases [10]. Lymph node metastasis occurs in approximately 14%-38% of UCS. Pelvic lymph node metastasis of UCS in more than half of patients shows para-aortic lymph node involvement [11]. In addition, a recent analysis of surveillance, epidemiological, and end-outcome data from stage I-III UCS showed that lymphadenectomy was associated with increasing survival compared to patients who did not undergo lymphadenectomy [12]. Therefore, pelvic and para-aortic lymph node dissection and greater omentectomy are recommended for UCS in the early stage. On the other hand, in patients with EC, pelvic and para-aortic lymphadenectomy are recommended only for moderate to high-risk disease [13]. The selection of anticarcinogen is also different for UCS and EC, and hormonal therapy is not applied to UCS [14, 15]. Due to the differences in their treatment methods and prognoses, the differential diagnosis of UCS and EC is essential.

Pre-operative diagnosis of UCS is suggested by imaging and is done through endometrial sampling. However, UCS is often diagnosed after hysterectomy because there is insufficient sensitivity in endometrial sampling to identify UCS (23.5-58.8%) [16, 17]. For the preoperative assessment of uterine carcinomas or sarcomas, magnetic resonance imaging (MRI) is frequently employed [18]. Therefore, MRI may play an important role to distinguish the two types of uterine tumor. Some authors described MR observations of hemorrhage, necrosis, and exophytic mass on USC [5], they also mentioned that T1-weighted images (T1WI) and T2-weighted images (T2WI) might be difficult to distinguish between UCS and EC. In addition, the conventional MRI findings of UCS were non-specific and could not differentiate them from certain EC [2, 3].

The prospective surgery or therapeutic therapy of these individuals depends on the preoperative diagnosis, differential diagnosis, and staging. Therefore, it is very meaningful to analyze the imaging characteristics of UCS. The lengthy process and limited resources involved in pathological biopsy would provide biased results. MRI examination is a good supplement. The purpose of our study was to analyze the clinical and imaging features of UCS and endometrial adenocarcinoma (EAC, the most common pathological type of EC), and to explore the diagnostic and the differential diagnostic accuracy using multiparametric MRI.

## Materials and methods

This study was observational and retrospective. All procedures in this study were approved by the Medical Ethics Committee of the First Affiliated Hospital of Fujian Medical University and the requirement for written informed consent was waived.

### Patients

Patients with pathologically proven UCS or EAC from January 2014 to June 2022 were identified from our hospital. Patients who met the following criteria were included in the study: 1) those who preoperatively underwent standard pelvic MRI examinations including T2WI, T1WI, diffusion-weighted imaging (DWI), apparent diffusion coefficient (ADC), contrast-enhanced (CE)-T1WI on the arterial and venous phases; 2) those with pathologically proven UCS or EAC after surgical resection. Patients who met the following criteria were excluded: 1) history of chemotherapy or radiation treatment before MRI examination; 2) those with poor image quality due to effect of artifact. Thus, a total of 51 patients were enrolled for analysis, including 17 UCS and 34 EAC patients.

### Clinical and pathological features

The clinical and pathological features were recorded in our study, including age, premenopause or postmenopause, clinical manifestation, the level of CA125, pathological pattern, FIGO stage, lymphatic metastasis, lymph-vascular space invasion (LVSI).

### MRI techniques

A 3.0 T MR scanner was used for every examination (MAGNETOM Verio, Siemens Healthineers). The standard dedicated pelvic MRI protocol consisted of the following sequences, transverse volumetric interpolated breath-hold examination with fat-suppression (VIBE)-T1WI, transverse and sagittal turbo spin echo with fat-suppression (TSE)-T2WI, and DWI (b value=50 and 800s/mm^2^). For the arterial and venous phases, CE-T1WI was performed in the transverse and sagittal planes at 40-60 seconds and 90-110 seconds, after intravenous injections of gadobenate dimeglumine (MultiHance, Bracco, 0.2mmol/kg body weight, rate of 3.0mL/s). The MRI protocol is shown in Table 1.

**Table 1:** MRI protocol

Sequences	TR (ms)	TE (ms)	Slice Thickness (mm)	Intersection Gap (mm)	FOV (mm)	Matrix
TSE-T2WI-SAG	4000	96	5	1.0	360 x 360	384 x 384 x 70%
TSE-T2WI-TRA	3400	91	5	1.0	240 x240	320 x320 x70%
DWI	6000	58	5	1.0	400 x 300	180 x 180 x 85%
VIBE-T1WI-TRA	3.2	1.2	3	0	360 x 300	384 x 384 x 70%
VIBE-T1WI-SAG	3.8	1.41	3	0	280 x280	320 x320 x70%

### Measurement of MRI values

All MRI were retrospectively reviewed by two radiologists with 5 and 15 years of experience in pelvic MRI, respectively, who were blinded to the clinical and the pathologic features (either UCS or EAC).

Image findings assessed included tumor size (in anterior-posterior, longitudinal, and transverse dimensions) on the sagittal and transverse T2WI, the boundary was clear or unclear, presence or absence of tumor cystic degeneration and necrosis or hemorrhage, the signal intensity (SI) of the tumor was homogeneous or heterogeneous on T2WI, invasion the depth of the myometrium, adjacent tissue invasion, lymph node metastasis, FIGO stage, the presence or absence of the feeding artery, the degree of enhancement, whether there were areas of strong enhancement. Cystic degeneration or necrosis was defined as areas of high SI on T2WI without enhancement after administration of contrast medium. Hemorrhage was defined as areas of high SI on T1WI.

**Fig. 1:**
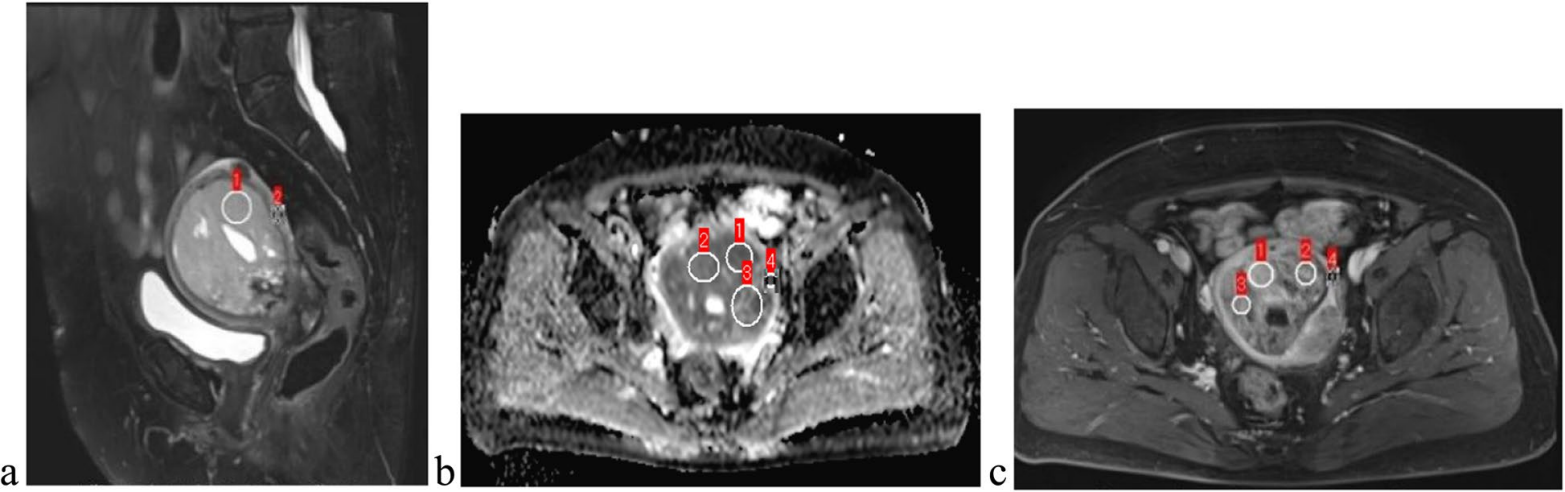
showed how the ROIs were selected and measured

We measured both tumor regions of interest (ROIs) and normal myometrium of uterus. Figure 1 showed how the ROIs were measured on T2WI (a), ADC (b), CE-T1WI on the arterial phase (c), respectively. The tumor ROI was placed as a single ROI at the level where the largest lesion could be measured on T2WI, T1WI, DWI. The maximum, mean, minimum ADC values (10^–3^mm^2^/s) and CE-T1WI on the arterial and venous phases were measured in a circular ROI in the representative location as large as possible within the tumor (ADCmax, ADCmean, ADCmin, REAmax, REAmean, REAmin, REVmax, REVmean, REVmin, respectively). The ROIs were placed on solid portion of the tumors to avoid necrosis, cystic degeneration, or hemorrhage as much as possible by reference to all MRI sequences including T2WI, T1WI, CE-T1WI, DWI. The normal myometrium of uterus ROIs were measured on the same slice of the lesion. And three ROIs were measured and then averaged for all of the parameters.

#### Calculated

The ratio of the tumor to the myometrium of uterus on T2WI, T1WI, DWI, ADC, CE-T1WI was calculated as follows: RSI (the relative signal intensity) = the signal intensity of tumor/normal myometrium of uterus (RSI-T2, RSI-T1, RSI-DWI, RSI-ADCmax, RSI-ADCmean, RSI-ADCmin); RE (the relative enhancement signal intensity) = (the contrast-enhanced SI of tumor - the unenhanced SI of tumor)/the contrast-enhanced SI of normal myometrium of uterus; The ROIs were drawn on the most, the average and the lowest enhancing component of the tumor on the contrast-enhanced T1WI obtained at the arterial and venous phases (REAmax, REAmean, REAmin, REVmax, REVmean, REVmin). REA=RE on thearterial phase, REV=RE on the venous phase.

### Statistical analysis

Categorical variables were described as frequencies and percentages. Continuous variables were described as means and standard deviations or medians and quartiles. The kappa statistic of concordance was used to assess inter-observer agreement. Kappa scores of 0.00-0.40, 0.4-0.60, 0.6-0.80, and >0.80 were regarded as poor, moderate, good, and excellent agreement, respectively. The X^2^ test was used to compare categorical characteristics between disease groups. Differences between continuous data were tested for significance using an independent t test. Multivariate logistic regression model was used to distinguish UCS from EAC using these characteristics. Receiver operating characteristic (ROC) analysis was used to assess the model’s performance, and the area under the curve (AUC) was used to evaluate the ability of prediction. The Youden index of sensitivity and specificity was used to determine the optimal cut-of values for UCS. All tests were two-sided, and p values of 0.05 or less were considered statistically significant. Statistical analysis was carried out using SPSS version 20.0.

## Results

### Clinical characteristics

The preoperative clinical features of the 51 cases were reviewed (Table 2). The mean age of UCS patients was higher than that of EAC (*p*<0.001). There were only 1 (5.9%) premenopausal woman with UCS and 15 (44.1%) premenopausal women with EAC (*p*<0.001). 13 (76.5%) patients with UCS and 28 (82.4%) patients with EAC suffered from abnormal vaginal bleeding, there was no statistical difference between the two groups (*p*=0.325). The level of CA125 was not significantly different between the two diseases (*p*=0.579).

**Table 2:** Clinical and pathological features of UCS and EAC

Characteristic	UCS (*n*=17)	EAC (*n*=34)	*p* value
Age, mean (range)	63.24±2.93	54.82±4.49	<0.001*
Postmenopausal			0.001*
Yes	16	19	
No	1	15	
Clinical presentation, no.			0.325
Abnormal vaginal bleeding	13	28	
Pelvic pain	3	6	
Abdominal mass	1	0	
CA125			0.579
Elevated	6	8	
normal	11	26	
Invasion depth			0.426
≤1/2 myometrium	8	20	
> 1/2 myometrium	9	14	
FIGO stage, no. (%)			0.214
I	9	20	
II	2	6	
III	6	8	
IV	0	0	
lymphatic metastasis			0.904
Yes	3	8	
No	14	26	
LVSI			0.692
Yes	8	18	
No	9	16	
UCS subtype, no. (%)			-
Homologous	4(23.53%)	-	
Heterologous	9(52.94%)	-	
Unspecified	4(23.53%)	-	
EAC classification, no. (%)			-
Grade 1	-	6(17.65%)	
Grade 2	-	10(29.41%)	
Grade 3	-	18(52.94%)	

### Pathological features

UCS and EAC did not significantly differ in depth of myometrial invasion (*p*=0.426) and FIGO stage (*p*=0.214) on pathology. Lymphatic metastasis and LVSI were also similar between the two disease groups (*p*=0.904, *p*=0.692, respectively). Of UCS cases, 52.94% were heterologous, 23.53% were homologous, and the rest 23.53% were an unspecified subtype. The proportion of grade 1-3 in the EAC cases was 17.65%, 29.41%, 52.94%, respectively (Table 2).

### MRI characteristics

**Fig. 2:**
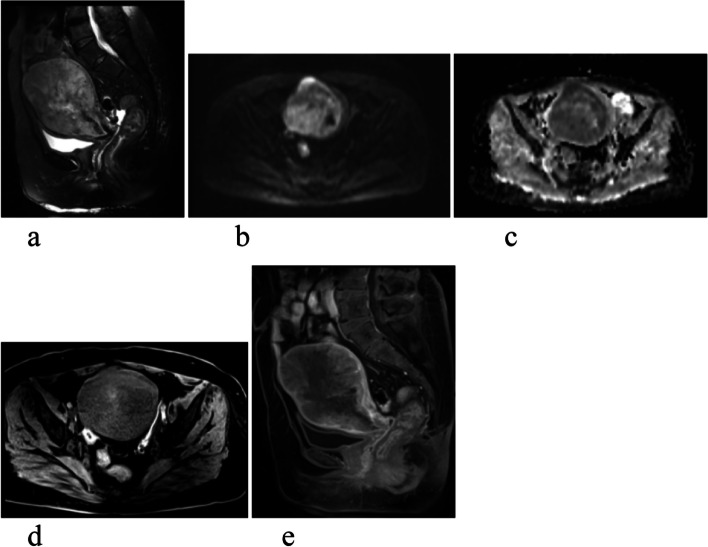
Images of uterine carcinosarcoma (UCS). **a** Sagittal T2WI of a patient shows a large intrauterine mass with heterogeneous high signal intensity. **b** Diffusion-weighted imaging (DWI) shows a high signal intensity tumor. **c** Apparent diffusion coefficient (ADC) displays mainly a low signal intensity tumor, some parts of high signal intensity. **d** Axial T1WI shows intratumoral hemorrhage of high signal intensity area in the mass. **e** Sagittal contrast enhanced T1WI shows a heterogeneous medium enhancement tumor, there is partial area without enhancement in the lesion

**Fig. 3:**
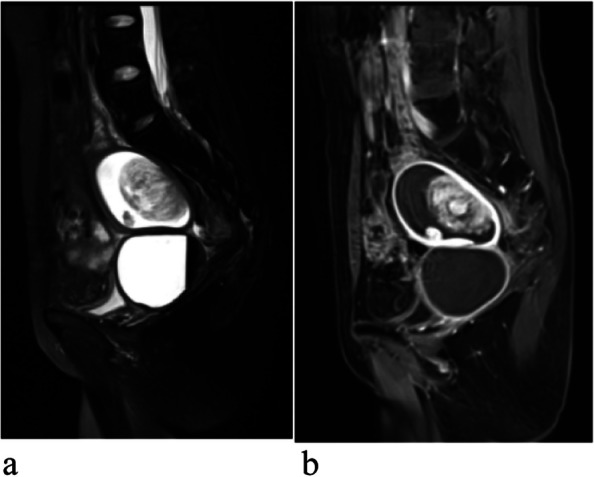
Images of UCS. **a**, **b** Sagittal T2WI of a patient shows a well-defined lesion with heterogeneous high signal intensity. **b** Sagittal contrast enhanced T1WI, there is an area in the mass showing strong enhancement similar to the myometrium

**Fig. 4:**
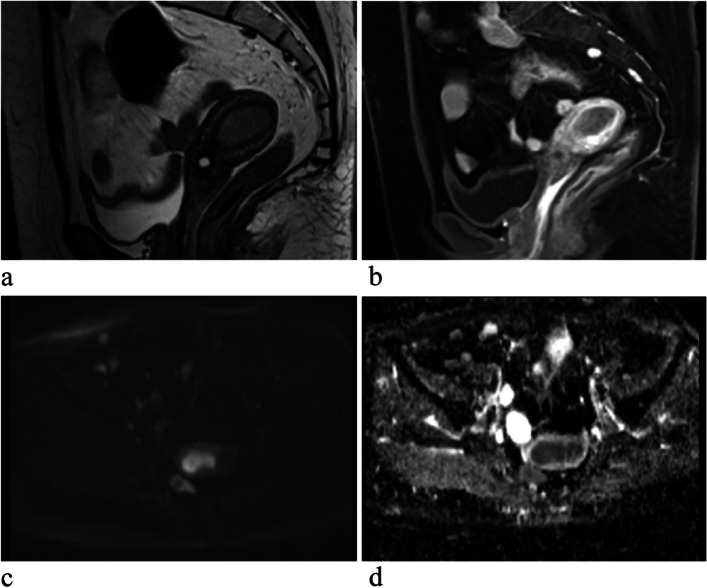
A patient with endometrioid adenocarcinoma (EAC). **a** Sagittal T2WI shows a homogeneous and slightly higher signal tumor. **b** Sagittal contrast enhanced T1WI, there is homogeneous mild enhancement in the mass lower than the myometrium. **c** DWI shows a obvious high signal intensity tumor. **d** ADC displays homogeneous low signal intensity in the tumor

**Fig. 5:**
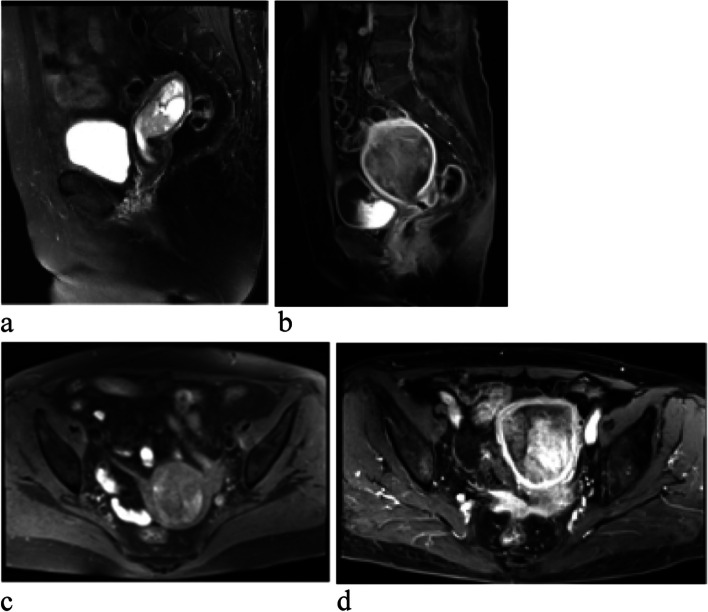
Images of UCS. **a** Sagittal T2WI shows a clear boundary and mixed signal tumor with high signal intensity of cystic degeneration or necrosis. **b** Sagittal contrast enhanced T1WI shows the feeding artery in the tumor. **c** Axial T1WI shows high signal intensity of hemorrhagein the mass. **d** Axial contrast enhanced T1WI, the tumor shows heterogeneous enhancement partly stronger than the myometrium in the tumor

**Fig. 6:**
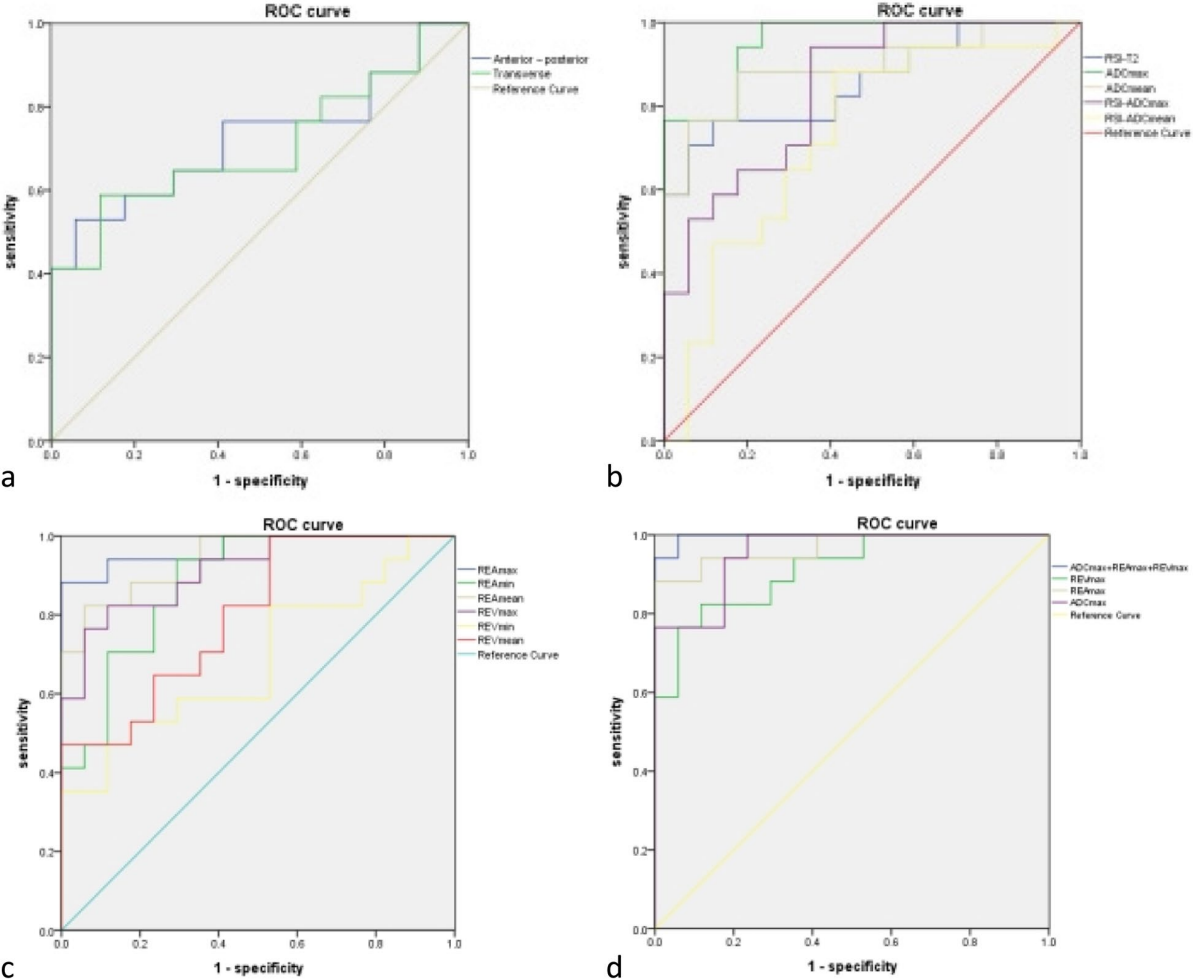
Receiver operating characteristic (ROC) curves show the threshold values of the MRI parameters for differentiating UCS from EAC. **a** The areas under the curve (AUCs) are 0.72, 0.71 for anterior-posterior and transverse dimensions, respectively. **b** The AUCs are 0.86, 0.96, 0.89, 0.84, 0.73 for RSI-T2, ADCmax, ADCmean, RSI-ADCmax, RSI-ADCmean, respectively. **c** The AUCs are 0.97, 0.88, 0.94, 0.91, 0.69 and 0.80 for REAmax, REAmin, REAmean, REVmax, REVmin, REVmean, respectively. **d** The AUC is 0.997 of the combined of ADCmax, REAmax and REVmax

Kappa statistics showed that the two readers reached the different agreements for MRI characteristics between UCS and EAC (Table 3). Table 3 summarized the MRI characteristics of the 17 UCS (Figs. 2 and 3) and 34 EAC (Fig. 4) cases. The anterior-posterior and transverse dimensions were significantly larger in UCS than EAC (*P*=0.011, *P*=0.015, respectively). However, there was no significant difference between UCS and EAC in longitudinal dimension (*P*=0.077). The boundary that was clear or unclear was not obviously different in the UCS and EAC (*P*=0.910). The cystic degeneration or necrosis and intratumoral hemorrhage were more likely occurred in UCS than EAC patients (*P*<0.001, *P*<0.001, respectively) (Fig. 5). Regarding the SI of T2WI, our study demonstrated that UCS were more heterogeneous than EAC (*P*<0.001) (Fig. 5). The tumors of UCS had higher RSI-T2 than that of EAC (*P*<0.001), however, there were no significant difference between UCS and EAC on RSI-T1, RSI-DWI (*P*=0.202, *P*=0.771, respectively). Both UCS and EAC showed low or equal SI on T1WI and high SI on DWI. Apart from that, ADCmax, ADCmean, RSI-ADCmax and RSI-ADCmean were significantly higher in UCS than EAC (*P*<0.001, *P*<0.001, *P*<0.001, *P*=0.019, respectively). While ADCmin and RSI-ADCmin were not statistical difference in the two disease groups (*p*=0.753, *p*=0.106,respectively). The REAmax, REAmin, REAmean, REVmax, REVmin and REVmean of UCS were all higher than that of EAC. The feeding artery and areas of strong enhancement were more likely to appear in UCS than EAC (*P*=0.021) (Fig. 5). Multivariate logistic regression model showed that there were not significant predictors of UCS. Figure 6 showed ROC results for significant features. The AUCs were 0.72, 0.71, 0.86, 0.96, 0.89, 0.84, 0.73, 0.97, 0.88, 0.94, 0.91, 0.69 and 0.80 for the anterior-posterior dimention, transverse dimention, RSI-T2, ADCmax, ADCmean, RSI-ADCmax, RSI-ADCmean, REAmax, REAmin, REAmean, REVmax, REVmin and REVmean, respectively. The AUC was 0.997 of the combined of ADCmax, REAmax and REVmax (Table 4). Our study showed that ADCmax threshold value of 789.05 (10^–3^mm^2^/s) could differentiate UCS from EAC with 100% sensitivity, 76.5% specificity, and 0.76 AUC. And the REAmax threshold value of 0.45 could differentiate UCS from EAC with 88.2% sensitivity, 100% specificity, and 0.88 AUC.

**Table 3:** MRI characteristics of UCS and EAC

	UCS (*n*=17)	EC (*n*=34)	Kappa value	*p* value
Tumor dimension				
Anterior–posterior	4.11±2.36	2.43±0.87	0.88(0.81-0.98)	0.011*
Longitudinal	6.37±3.67	4.61±1.77	0.87(0.80-0.97)	0.077
Transverse	4.97±2.72	3.15±0.98	0.90(0.79-0.96)	0.015*
Boundary			0.79(0.73-0.93)	0.910
Unclear	5	8		
Clear	12	26		
Cystic degeneration or necrosis			0.80(0.71-0.89)	<0.001*
Yes	11	0		
No	6	34		
Hemorrhage			0.82(0.74-0.95)	<0.001*
Yes	8	2		
No	9	33		
Signalintensity on T2WI			0.85(0.78-0.96)	<0.001*
homogeneous	4	32		
heterogeneous	13	2		
RSI-T2(mean ± SD)	1.97±0.47	1.41±0.32	0.73(0.63-0.81)	<0.001*
RSI-T1	0.87±0.09	0.93±0.18	0.71(0.60-0.78)	0.202
RSI-DWI	2.76±0.57	2.72±0.49	0.69(0.58-0.76)	0.771
ADCmax	1166.18±325.37	752.35±81.22	0.65(0.53-0.82)	<0.001*
ADCmin	598.23±145.71	608.94±94.47	0.74(0.62-0.84)	0.753
ADCmean	904.85±187.24	672.06±86.54	0.67(0.59-0.91)	<0.001*
RSI-ADCmax	0.71±0.15	0.53±0.12	0.62(0.49-0.72)	<0.001*
RSI-ADCmin	0.37±0.11	0.43±0.11	0.59(0.38-0.65)	0.106
RSI-ADCmean	0.55±0.10	0.47±0.11	0.66(0.53-0.81)	0.019*
REAmax	0.69±0.27	0.24±0.10	0.58(0.39-0.75)	<0.001*
REAmin	0.31±0.13	0.11±0.10	0.55(0.47-0.78)	<0.001*
REAmean	0.44±0.14	0.17±0.09	0.62(0.54-0.82)	<0.001*
REVmax	0.64±0.25	0.29±0.13	0.61(0.43-0.77)	<0.001*
REVmin	0.29±0.13	0.21±0.12	0.54(0.38-0.79)	0.031*
REVmean	0.42±0.16	0.24±0.12	0.67(0.40-0.84)	0.001*
Feeding artery			0.85(0.73-0.92)	0.021*
Yes	6	2		
No	11	32		
Areas of strong enhancement			0.83(0.69-0.88)	<0.001*
Yes	13	0		
No	4	34

**Table 4:** ROC curve

parameters	AUC	*P* value	95% CI
Anterior-posterior dimension	0.723	0.010*	0.555-0.892
Transverse dimension	0.706	0.017*	0.535-0.877
RSI-T2	0.858	<0.001*	0.739-0.977
ADCmax	0.955	<0.001*	0.892-1.000
ADCmean	0.893	<0.001*	0.786-0.999
RSI-ADCmax	0.841	<0.001*	0.732-0.949
RSI-ADCmean	0.727	0.009	0.580-0.873
REAmax	0.969	<0.001*	0.000-1.000
REAmin	0.882	<0.001*	0.793-0.972
REAmean	0.941	<0.001*	0.869-1.000
REVmax	0.913	<0.001*	0.831-0.996
REVmin	0.689	0.029*	0.523-0.854
REVmean	0.799	0.001*	0.674-0.925
ADCmax+REAmax+REVmax	0.997	<0.001*	0.000-1.000

*CI* Confidence interval

## Discussion

UCS is misdiagnosed as EC frequently at that time due to a lack of pathological specimens. A research found that 75% of UCS patients were preoperatively mistakenly diagnosed as EC [2]. UCS is typically diagnosed correctly depending on ultimate pathological findings following surgical resection [19]. UCS has an aggressive clinical course and a poor overall prognosis. Even if UCS is in stage I, the 5 year survival rate is still less than 50%. The typical clinical symptom is abnomal vaginal bleeding, other symptoms include abdominal mass, abdominal pain. However, these symptoms are not specific. Although both UCS and EC can be treated by surgery, there are still significant differences in the surgical methods. For EC, the standard surgery is hysterectomy and bilateral adnexectomy , and with or not lymph node dissection [20]. In the EAC FIGO IA G1-G2 disease, lymph node could not be evaluated since the risk of nodal metastasis is fairly low (<5%) [21]. The primary treatment for non-metastatic UCS is complete surgical staging, including total hysterectomy, salpingo-oophorectomy, and lymph node staging [22]. In addition, staged excision of omentum should be considered for UCS [22]. Therefore, uterine tumor imaging may play an important role if it can help the clinician make a correct diagnosis early on, before the operation.

According to histology, UCS is composed of epithelial and mesenchymal components. UCS is classified as heterologous or homologous according to the sort of cells that make up the sarcomatous component. Heterologous types include rhabdomyosarcoma, chondrosarcoma, osteosarcoma, or liposarcoma, and homologous types include fibrosarcoma, endometrial stromal sarcoma, or leiomyosarcoma. In either situation, the carcinomatous component may be made up of endometrioid, serous, or clear cell types. Recent investigations in immunohistochemistry, ultrastructure, and molecular biology have all pointed to carcinosarcomas being metaplastic carcinomas, with the mesenchymal component typically retaining at least some epithelial characteristics. As a result, some experts contend that UCS is better classified as a particular kind of EC. And cancerous components are the driving force for tumor progression. The risk factors and clinical manifestations of UCS are similar to those of EC [23].

Regard to the conspicuity of the tumor margin on T2WI, we predicted that UCS would reveal a clearer border between the tumor and myometrium because that was often a clearly defined exophytic mass [24]. However, there was not significant difference in this respect between UCS and EAC. This is probably due to the fact that MRI has a higher resolution of soft tissue and can clearly distinguish endometrial, myometrial and interstitial limits. Previous studies had shown that UCS tended to appear as larger, heterogeneous tumors with deep myometrial invasion unlike EC, but that were not specific and could be similar with invasive EC [25]. In our study, 76.47% of UCS presented with mixed SI on T2WI, which was consistent with the complicated histopathological components. UCS has a combination of cancer and sarcoma, and sometimes even various sarcomas. The heterogeneous SI on T2WI has also been described as a feature of the UCS [2]. Hemorrhage, cystic degeneration or necrosis is common, which may lead to the heterogeneous SI of UCS on T2WI. EAC almost shows homogeneous SI on T2WI. Therefore, homogeneous SI on T2WI is a reliable indicator to distinguish between UCS and EAC. Our study also demonstrated that UCS had higher RSI-T2 than EAC, that was in good agreement with previous reports [24].

DWI is gradually recognized in body imaging for the identification of malignancies, and ADC values have been used to describe tumor functions [26–28]. DWI is a well-known method for finding uterine tumors and offers a large tissue contrast to evaluate the extent of muscle infiltration by these tumors [29, 30]. Several recent reports have generally used ADC values obtained in uterine imaging to differentiate benign tumors from malignant ones [31, 32]. UCS that contains cartilage, nerve, calcification, necrosis, and hemorrhage would have high ADC values for component diversity. It was reported that the ADCmean of UCS was much higher than that of grade 2 or 3 EC [19]. Furthermore, the ADC map could also make a distinction between adenocarcinoma and sarcoma [33], since the ADC map was more heterogeneous in the sarcomatous component [34]. High ADC values have been reported to indicate high-grade malignancy with necrosis, which was often more common with UCS than EC [19]. It was similar with our findings that the ADCmax, ADCmean, RSI-ADCmax and RSI-ADCmean of UCS were significantly higher than EAC. The result may possibly reflect the tissue heterogeneity of UCS including abundant microscopic necrotic regions and epithelial cystic components, which could increase the ADC values. Previous research on EC had demonstrated that significant difference in ADC values that help distinguish between histological grades, with high-grade tumors producing low ADC values and low-grade tumors producing high ADC values [31, 35].

UCS was enhanced equally or more strongly than uterine myometrium, and more strongly than EC, in good agreement with previous reports [2, 24]. Takemori et al. [36] showed that the sarcomatous component was enhanced more strongly than the carcinomatous component on contrast-enhanced T1WI, because the sarcomatous component had substantial vascularity. This may explain,that UCS was more likely to occur the feeding artery and areas of strong enhancement than EAC. Ohguri et al. [2] also reported that all four UCS showed areas of early and persistent marked enhancement similar to that of uterine myometrium and found that the portions with high SI in the early phase dynamic study corresponded histologically to sarcomatous components with prominent vascularity.

Matsuo et al. [37] reported that not only did the carcinoma component play a major role in tumor progression and survival, but also the sarcoma component make a significant contribution. The assumption is that tumor necrosis arises from chronic ischemic lesions due to the rapid growth of tumors. [38]. Hypoxia is frequently present in necrosis, which causes the activation of hypoxia-inducible factors [39]. Ischemic regions lead to tumor progression by promoting overexpression of hypoxia-inducible factors under hypoxic circumstances [40]. These results indicate that non-enhanced areas caused by necrosis probably reflect a highly aggressive tumor with active proliferation.

As EC was homogeneously enhanced lower than the myometrium in general, they observed that different contrast-enhanced patterns inside an endometria tumor may increase the possibility of UCS. As a result, different patterns of enhancement within an endometrial mass may represent a mixture of different histopathology. UCS should be distinguished from EAC because their treatment strategy and prognosis are different [41]. However, the histologic results are sometimes misleading, as the biphasic nature may sometimes not be apparent until the entire tumor is investigated. Tanaka et al. [24] reported that UCS mainly had strongly enhanced regions and unenhanced areas on T1WI within the mass. Therefore, highly enhanced areas can predict the possibility of UCS to diagnose a malignant tumor of the endometrium.

Other studies had shown that UCS generally displayed early hyper-enhancement relative to the myometrium persisting into the delayed phase, whereas EC more frequently had hypo-enhancement relative to the myometrium [42]. These were similar to our study that UCS was associated with higher enhancement during the arterial and venous phases compared to EAC. Emoto and colleagues reported that UCS had greater angiogenic activity than EC due to over-expression of VEGF in cancer cells and expression of the Ang-2 gene at the periphery of the tumour [43]. As previously published data had demonstrated that conventional contrast-enhanced MRI can not distinguish UCS from EC, we believe that the unique ability and higher diagnostic accuracy of semiquantitative parameters of the REAmax, REAmin, REAmean, REVmax, REVmin and REVmean to differentiate UCS from EAC will have a significant impact in clinical practice. UCS often showed progressive or persistent enhancement, while EAC often showed mild enhancement [44]. This was another important differential point to distinguish UCS from EAC. Our study showed that ADCmax threshold value of 789.05 (10^–3^mm^2^/s) could differentiate UCS from EAC with 100% sensitivity, 76.5% specificity, and 0.97 AUC. And the REAmax threshold value of 0.23 could differentiate UCS from EAC with 94% sensitivity, 88% specifcity, and 0.97 AUC. The ROC curve has been widely applied in the evaluation of radiologic imaging, and diagnostic accuracy is characterized by the combination of sensitivity and specificity. Relatively higher ADCmax values and strongly enhanced areas may predict the possibility of UCS to EAC.

There are several limitations to our study. First, this is a retrospective study using a small sample size of 17 patients because of the rarity of UCS and therefore subject to potential selection bias. A larger sample of data is needed to confirm these findings. Second, studies were not all carried out on the same kind of MRI scanner, and there were some variations in protocol. Third, because some instances lacked the necessary sequences, we were unable to assess the value of MR spectroscopy or perfusion imaging. Fourth, we did not contrast the MRI findings with the pathological features. Future research should use radiological pathological correlation to validate the imaging characteristics of UCS identified in this study.

In conclusion, UCS was more common in postmenopausal patients and the main manifestation was abnormal vaginal bleeding. The SI on T2WI was more heterogeneous in UCS than EAC. Based on semiquantitative characteristics and the enhancement pattern, MRI may be utilized as a biomarker to distinguish UCS from EAC, which may help with appropriate preoperative characterisation and therapy stratification in these individuals. These results need to be confirmed by prospective studies.

## Data Availability

The datasets used during the current study are available from the corresponding author on reasonable request.
